# Influence of Gut Microbiota on Response to Immune Check Point Inhibitors in MASLD Patients With HCC: Unraveling the Connection

**DOI:** 10.1002/cam4.71738

**Published:** 2026-03-27

**Authors:** Mazen Elsheikh, Mohamad Ali Ibrahim, Sherry Fares, Megha Bhongade, Karim Adhem, Ximena I. Ramirez‐Morales, Ahmed O. Kaseb, Joseph Petrosino, Manal M. Hassan, Prasun K. Jalal

**Affiliations:** ^1^ Section of Gastroenterology and Hepatology Baylor College of Medicine Houston Texas USA; ^2^ Division of Cancer Medicine, Department of Gastrointestinal Medical Oncology The University of Texas, MD Anderson Cancer Center Houston Texas USA; ^3^ Department of Molecular Virology and Microbiology, Alkek Center for Metagenomics and Microbiome Research Baylor College of Medicine Houston Texas USA; ^4^ Division of Cancer Prevention and Population Sciences, Department of Epidemiology The University of Texas, MD Anderson Cancer Center Houston Texas USA

**Keywords:** dysbiosis, fecal microbial transplantation, immune checkpoint inhibitors

## Abstract

Immune checkpoint inhibitors (ICIs) have emerged as a promising treatment for various cancers, including advanced hepatocellular carcinoma (HCC). However, a significant proportion of patients with HCC, particularly those with metabolic dysfunction‐associated liver disease (MASLD), exhibit resistance to ICI therapy. Studies have revealed that the presence of specific gut bacteria, such as *Akkermansia*, *Bifidobacterium*, and *Lachnoclostridium*, is associated with improved outcomes with ICI‐treated HCC patients. Conversely, the overgrowth of bacteria like *Enterobacteriaceae* is linked to resistance to therapy. This review investigates the role of gut microbiota in shaping immune checkpoint inhibitor responses in MASLD‐related hepatocellular carcinoma, focusing on how dysbiosis may contribute to ICI resistance and exploring microbiome modulation strategies, such as fecal microbiota transplantation and probiotics, aiming to optimize therapeutic outcomes.

## 1 Introduction

Immune checkpoint inhibitors (ICIs) have emerged as an advanced approach for treating various types of cancer in humans [1, 2]. By unleashing the immune system's natural response, these groundbreaking treatments have expanded the array of choices for dealing with various cancers such as melanoma, breast cancer, liver cancer, and bladder cancer [3]. In advanced hepatocellular carcinoma (HCC), ICIs have proven effective as both first‐ and second‐line treatments [4]. However, while these drugs have revolutionized care for viral‐induced HCC, they frequently fail in patients with metabolic dysfunction‐associated liver disease (MASLD) HCC [5, 6].

This apparent paradox largely reflects fundamental differences in the immunologic contexture of these disease states. In viral hepatitis–associated HCC, the immune system can recognize viral antigens presented by infected hepatocytes, eliciting relatively robust virus‐specific CD8+ T‐cell responses [7]. Although the tumor microenvironment is immunosuppressive, a baseline level of functional, antigen‐specific immunity often persists. This underlying “viral surveillance” creates an immune landscape that is more readily reinvigorated by checkpoint blockade [8].

By contrast, MASLD‐HCC develops in a distinct immune milieu shaped by metabolic inflammation, an immunosuppressive tumor microenvironment, and dysbiosis‐driven immune dysfunction. Unlike viral HCC, the barrier to ICI efficacy in MASLD‐HCC is not simply checkpoint upregulation but rather a pre‐exhausted immune microenvironment in which the gut microbiota plays a mandatory in shaping antitumor immune competence and therapeutic responsiveness [5, 6, 9, 10, 11, 12].

## 2 Dysbiosis and MASLD‐HCC

Recent research has confirmed the significant role of dysbiosis in the pathogenesis of Metabolic Dysfunction‐Associated Steatohepatitis (MASH) and MASLD‐HCC, highlighting dynamic microbial changes during the transformation of MASH into MASH‐HCC [13, 14]. Research consistently shows that individuals with MASLD display unique patterns of gut dysbiosis when compared to healthy individuals, with these microbial imbalances becoming increasingly pronounced as the disease advances from simple steatosis to MASH, fibrosis, and ultimately hepatocellular carcinoma [15, 16].

**FIGURE 1:**
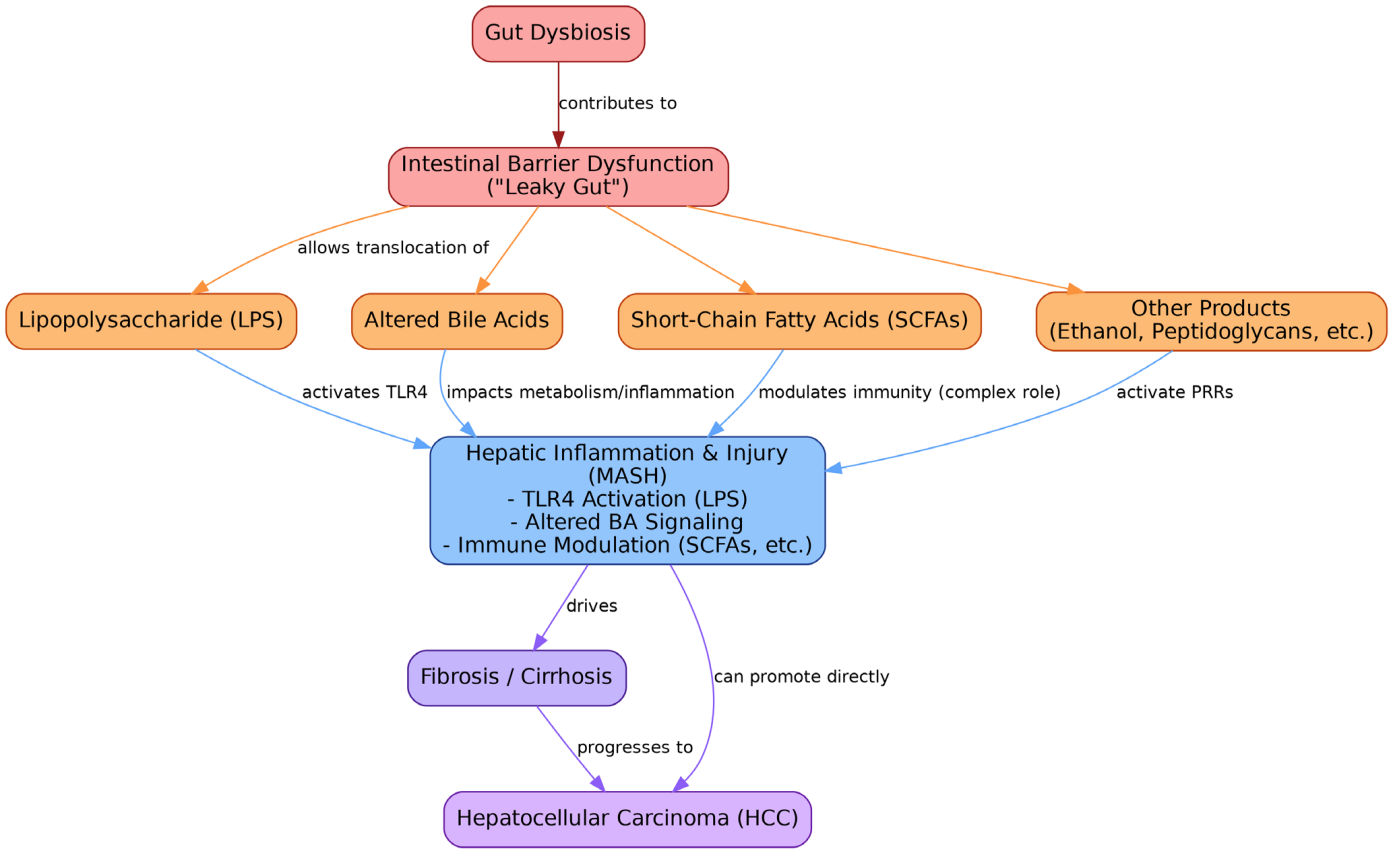
Key pathways linking gut dysbiosis to MASLD and MASLD HCC. HCC, hepatocellular carcinoma; LPS, lipopolysaccharide; MASH, metabolic dysfunction–associated steatohepatitis; MASLD, metabolic dysfunction–associated steatotic liver disease; PRRs, pattern recognition receptors; SCFAs, short‐chain fatty acids; TLR4, Toll‐like receptor 4.

Gut dysbiosis in MASLD leads to intestinal barrier disruption, increasing intestinal permeability that allows bacterial products like lipopolysaccharide (LPS) to translocate into the portal circulation, triggering hepatic inflammation [17, 18]. These translocated bacterial components activate TLR4 signaling in Kupffer cells, initiating proinflammatory cascades, which promote progression from steatosis to steatohepatitis and fibrosis [15, 19]. Additionally, dysbiotic gut microbiota alter microbial metabolite production, including increased ethanol synthesis, disrupted bile acid homeostasis, and reduced beneficial short‐chain fatty acids, which collectively contribute to hepatic lipid accumulation, oxidative stress, and the inflammatory microenvironment that promotes hepatocarcinogenesis [17, 20, 21]. Key pathways linking gut dysbiosis to hepatocellular carcinoma progression in MASLD are summarized in Figure 1.

Emerging evidence highlights the gut microbiome as a pivotal modulator of the immune system in HCC, influencing both disease progression and treatment efficacy. Specifically, gut dysbiosis has been found to impact HCC patients' response to ICI therapy. Research indicates that HCC patients who exhibit positive responses to ICIs typically harbor a favorable gut microbiota profile and reduced levels of intestinal inflammation [22].

The specific bacterial signatures of these responders vary by disease etiology: in viral HCC, beneficial responses are linked to an enrichment of *Bifidobacterium* and *Faecalibacterium*, while in MASLD‐HCC, responders are characterized by the presence of *Mediterraneibacter gnavus* [23]. Beyond specific bacteria, certain microbial metabolites also appear to enhance ICI efficacy [24, 25]. Importantly, this relationship is bidirectional; ICI treatment itself has been shown to alter gut microbial composition, suggesting a dynamic interplay between the therapy and the intestinal ecosystem [22, 24, 26]. These key associations between microbial taxa and treatment outcomes are summarized in Table 1.

**TABLE 1:** Studies suggesting association between gut microbiota population and ICI response in HCC patients.

Study	Enrolled patients number	Detection method	Intervention type	HCC etiology	Tumor response evaluation criteria used	Potential response biomarkers	Shift in microbial composition during ICI treatment
Zheng et al. (2019) [26]	8	Metagenomic sequencing	Camrelizumab	NA	RECIST 1.1 [27]	R: *Akkermansia muciniphilia* and *Ruminococcaceae* NR: *Proteobacteria*	Yes
Li and Ye (2020) [28]	65	16S rRNA sequencing	ICIs (unspecified)	HBV patients (100%)	RECIST 1.1 [27]	R: *Ruminococcaceae, Faecalibacterium* Extended PFS: *Faecalibacterium* NR and shorter PFS: *Bacteroidales*	—
Chung et al. (2021) [4]	8	16S rRNA sequencing	Nivolumab	Mixed	RECIST 1.1 [27]	R: *Azospirillum* sp., *Citrobacter freundii* , and *Enterococcus durans* R: High *Prevotella* to *Bacteroides* species ratio NR: *Dialister pneumosintes* , *Enterococcus faecium* , *Escherichia coli* , *Granulicatella* sp., *Lactobacillus reteri*, *Streptococcus gordonii* , *Streptococcus mutans* , *Trichuris trichiura*, and *Veillonella atypica* NR: Imbalanced *Firmicutes* to *Bacteroidetes* ratio (< 0.5 or > 1.5)	—
Wu et al. (2022) [24]	35	16S rRNA sequencing	Anti‐PD‐1 treatment	Mixed	mRECIST [29]	R: *Blautia, Coprococcus, Dorea, Faecalibacterium, Haemophilus, Lachnospiracea incertae sedis, Megamonas*, and *Ruminococcus* R: Serum Alpha‐D Glucose metabolite NR: *Allisonella, Atopobium, Bifidobacterium, Campylobacter, Lactobacillus, Leptotrichia, Methanobrevibacter*, and *Parabacteroides*	Yes
Lee et al. (2022) [25]	41	16S rRNA sequencing	Nivolumab and pembrolizumab	Mixed	RECIST 1.1 [27]	R: *Lachnoclostridium, Lachnospiraceae*, and *Veillonella*, Fecal Ursodeoxycholic acid and ursocholic acid Improved OS: *Lachnoclostridium* NR, declined OS: Prevotella 9	—
Ponziani et al. (2022) [22]	11	16S rRNA sequencing	Tremelimumab and/or Durvalumab	Mixed	mRECIST [29]	R: *Akkermansia* NR: *Enterobacteriaceae*, Fecal calprotectin concentrations, PD‐L1 serum levels	Yes
Zhu et al. (2024) [30]	80	Metagenomic sequencing	PD‐1/PD‐L1 inhibitors (toripalimab, camrelizumab, etc.) plus targeted therapy (lenvatinib 77.5%)	Mixed	RECIST 1.1 [27]	R: *Phascolarctobacterium faecium* , *Candidatus Avimonas narfia* NR: *Actinomyces_sp_ICM47, Senegalimassilia anaerobia, Faecalibacillus faecis*	Yes
Xin et al. (2024) [31]	45	Metagenomic sequencing	Anti‐PD‐1 combined with a tyrosine kinase inhibitor (TKI) plus a locoregional therapy	Mixed	mRECIST [29]	R: *Collinsella genus, Ruminococcus_AM4211, Ruminococcus_AF25_28AC* NR: *Bacteroides_AF20_13LB, Veillonella_atypica, Veillonella_AF13_2*	
Lee et al. (2025) [23]	Total: 102 MASLD‐HCC (*n* = 25) Viral‐HCC (*n* = 77)	16S rRNA sequencing	Atezolizumab+Bevacizumab (44%), Pembrolizumab+Lenvatinib (52%), Tremelimumab+Durvalumab (4%)	MASLD‐HCC only	RECIST 1.1 [27]	MASLD HCC R: Mediterraneibacter gnavus NR: *Kluyvera georgiana* , *Klebsiella oxytoca* , *Enterococcus faecium* 2 Viral HCC R: Bifidobacterium NR: Marseillibacter massiliensis, Vescimonas fastidiosa	—
Wu et al. (2025) [32]	53	Metagenomic sequencing	Anti‐PD‐1 treatment	Mixed: MASLD‐HCC (9 patients), Viral HCC only (20 patients)	RECIST 1.1 [27]	R: ↑ *Akkermansia muciniphila* NR: ↓ *Akkermansia muciniphila*	

Abbreviations: ALD, alcoholic liver diseases; MASLD, metabolic dysfunction‐associated liver disease; mRECIST, modified RECIST; NR, non responders; OS, overall survival; PFS, progression‐free survival; R, responders; RECIST 1.1, response evaluation criteria in solid tumors, version 1.1.

While the research results are promising, it is notable that accumulating evidence linking gut dysbiosis to ICI response in HCC reveals substantial heterogeneity in study designs, patient populations, and findings that necessitate critical appraisal. Several bacterial taxa emerge as robust biomarkers appearing consistently across multiple independent studies, thereby representing more generalizable findings. *Akkermansia muciniphila* , identified as an ICI responder‐associated bacterium in three separate studies [22, 26, 32], demonstrates the most compelling evidence for a conserved role in promoting immunotherapy response. Similarly, Ruminococcaceae species appear across four studies [24, 26, 28, 31], consistently associating with favorable ICI response, making them among the most reproducible microbiota signatures in HCC immunotherapy. Faecalibacterium and Lachnoclostridium also demonstrate multi‐study validation, appearing in at least two independent cohorts with consistent positive associations. In contrast, numerous bacterial taxa identified as either responder or non‐responder biomarkers derive from single‐study findings. Examples include *Phascolarctobacterium faecium* and Candidatus Avimonas narfia [30], Collinsella genus [31], and the species‐level organisms identified by Chung et al. [4], such as Azospirillum, *Citrobacter freundii* , and multiple Enterococcus and Streptococcus species. These bacteria, while potentially important, remain inadequately validated and their clinical significance cannot be confirmed without reproduction in larger, independent cohorts. Notably, certain bacteria exhibit conflicting associations across studies, with Bifidobacterium demonstrating opposing effects between HCC etiologies (enriched in responders in viral HCC subgroup, but not prominent in MASLD‐HCC subgroup responders) [23], Prevotella showing divergent outcomes between Chung et al. [4] and Lee et al. [25], and Veillonella appearing as both a responder and non‐responder marker depending on the study [4, 25, 31]. This critical finding highlights that etiology‐specific microbiota signatures exist. Importantly, Lee et al. [23] includes a properly stratified design comparing 25 MASLD‐HCC patients directly with 77 viral HCC patients using identical methodologies, revealing that Mediterraneibacter gnavus uniquely characterizes MASLD‐HCC responders while Bifidobacterium enrichment predicts favorable response in viral HCC. This represents the most direct evidence of etiology‐dependent microbiota‐ICI associations available. However, most other studies enrolled mixed HCC populations without stratifying patients by underlying liver disease etiology, making it difficult to discern whether reported bacterial associations reflect universal HCC biology or etiology‐specific phenomena masked by population heterogeneity. Sample size variation is also notable, ranging from 8 to 80 participants per study, which may influence statistical power to detect microbiota associations. Taken together, these observations illustrate that while current evidence is limited by mixed populations and variable sample sizes, a solid foundation of reproducible microbiota‐ICI associations has nevertheless emerged. The identification of consistent markers like *Akkermansia* and *Ruminococcaceae* confirms the biological relevance of this field. Moving forward, the variability observed across studies should not be viewed as a failure, but as a signal that precision microbiome profiling is necessary.

To understand how dysbiosis can influence ICI efficacy in MASLD‐HCC, we must first explore the mechanisms by which ICIs activate anti‐tumor immunity, setting the stage for examining their specific application in this disease.

## 3 Mechanism of Action of ICIs

**FIGURE 2:**
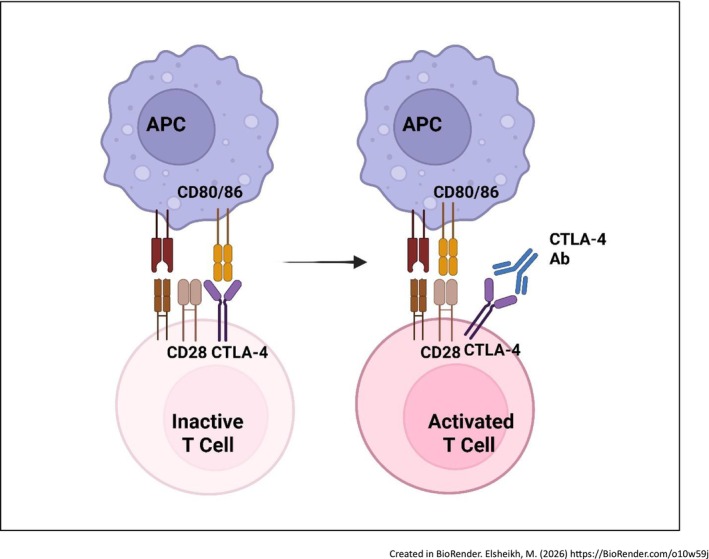
Mechanism of action of anti CTLA‐4 antibodies. APC, antigen‐presenting cell; CD, cluster of differentiation; CTLA‐4, cytotoxic T‐lymphocyte–associated protein 4.

**FIGURE 3:**
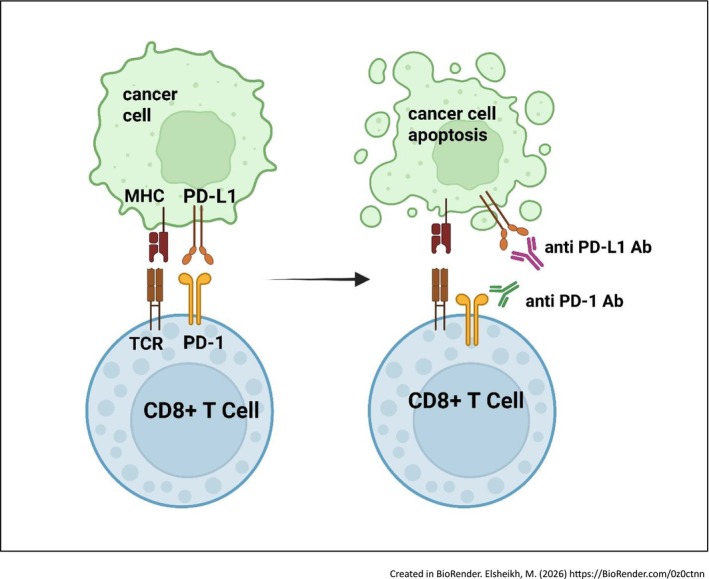
Mechanism of action of anti‐PD1/PDL1 antibodies. APC, antigen‐presenting cell; CD, cluster of differentiation; MHC, major histocompatibility complex; PD‐1, programmed cell death protein 1; PD‐L1, programmed death‐ligand 1; TCR, T‐cell receptor.

ICIs, including anti‐PD‐1 and anti‐CTLA‐4 antibodies, function by interfering with immune checkpoint mechanisms to boost T‐cell responses against cancer [33]. In normal immune surveillance, CD8+ cytotoxic T cells identify tumor antigens, penetrate tumors, and destroy malignant cells [34, 35, 36], however, inhibitory checkpoints such as CTLA‐4 and PD‐1 on T cells interact with their corresponding ligands (CD80/CD86 and PD‐L1/PD‐L2) on antigen‐presenting or tumor cells to dampen this response and prevent autoimmunity [37, 38]. Cancer cells frequently exploit these regulatory pathways to escape immune detection and destruction. By blocking these inhibitory interactions, ICIs effectively restore T‐cell cytotoxic activity against tumors, though this therapeutic mechanism can be influenced by external factors like the gut microbiome (Figures 2 and 3) [39].

## 4 ICIs in MASLD‐HCC

Despite the promising potential of ICIs in cancer treatment, the current response rate in various types of cancers stands at only 15%–20% [40]. While some cancer patients may derive benefits from ICI therapy, a significant portion either exhibit no response to the treatment or develop resistance following an initial positive reaction. Notably, HCC, particularly in patients with MASLD, has emerged as a cancer type demonstrating resistance to ICIs. Clinical data reveal that viral‐associated HCC patients achieve nearly double the objective response rates (38% vs. 16%) and significantly longer median overall survival (19.3 vs. 11.4 months) than MASLD HCC [41, 42].

A comprehensive study involving 130 HCC patients undergoing anti‐PD‐(L)1 immunotherapy revealed striking disparities in treatment outcomes [43]. Among the cohort, 13 patients were diagnosed with MASLD, while the remaining 117 patients had other underlying liver diseases etiologies. Remarkably, the MASLD subgroup exhibited a significantly decreased median overall survival post‐immunotherapy, spanning merely 5.4 months compared to 11.0 months for non‐MASLD patients (*p* = 0.023) [43]. This discrepancy was observed despite the MASLD HCC patients presenting with lower baseline rates of macrovascular tumor invasion (23% vs. 49%) and a higher frequency of receiving immunotherapy as a first‐line treatment (46% vs. 23%). Even after adjusting for potential prognostic confounders such as macrovascular tumor invasion, extrahepatic metastases, performance status, and alpha‐fetoprotein levels, MASLD was still independently linked to reduced survival in HCC patients following anti‐PD1 treatment (hazard ratio [HR] 2.6; 95% confidence interval [CI] 1.2–5.6; *p* = 0.017) [43]. The study findings underscore the concerning probability that individuals with underlying MASLD did not reap the benefits of checkpoint‐inhibition therapy [43].

In the pivotal IMbrave150 study, while atezolizumab plus bevacizumab established a new standard of care, subgroup analyses revealed a critical disparity: the combination did not demonstrate a statistically significant overall survival benefit compared to sorafenib in patients with non‐viral HCC. This stands in sharp contrast to the substantial survival advantage observed in viral‐associated case [44, 45].

Moreover, two meta‐analyses reinforce this, indicating that patients with MASLD‐HCC are unlikely to derive significant therapeutic benefit from ICIs when used as a standalone or primary modality [46, 47]. The first meta‐analysis, encompassing three global phase III trials (IMbrave150, KEYNOTE‐240, and CheckMate459) with 1656 patients, found that while ICIs significantly improved overall survival in viral HCC (HR: 0.64; 95% CI: 0.48–0.84), they failed to confer a survival advantage in non‐viral HCC (HR: 0.92; 95% CI: 0.77–1.11) [43]. Similarly, a second meta‐analysis of eight randomized controlled trials involving 3739 patients confirmed that the superiority of ICIs over standard care disappears in non‐viral etiologies [47]. Conversely, the efficacy of tyrosine kinase inhibitors or anti‐VEGF agents remains consistent regardless of etiology [47].

Significantly, recent evidence indicates that the negative impact of MASLD extends even to patients with viral etiologies. A retrospective study of 155 patients with chronic hepatitis B (CHB)‐related HCC treated with ICI‐based therapy demonstrated that concurrent MASLD significantly compromised treatment efficacy [5]. Patients with MASLD concurrent with CHB (MASLD‐CHB) exhibited significantly shorter median progression‐free survival (PFS) compared to those with CHB alone (6.9 months vs. 9.3 months; *p* = 0.001) [5]. Furthermore, the MASLD‐CHB group had a higher rate of progressive disease (57.89% vs. 37.61%; *p* = 0.028) and a lower disease control rate (42.11% vs. 62.39%; *p* = 0.028). Multivariate analysis identified concurrent MASLD as an independent risk factor for shorter PFS (HR 1.921; 95% CI 1.138–3.245; *p* = 0.015) [5]. Intriguingly, this resistance occurred despite the MASLD‐CHB group showing significantly higher percentages of CD4 + PD1+ and CD8 + PD1+ T cells in the tumor microenvironment (*p* < 0.001 and *p* = 0.005, respectively), suggesting a state of immune exhaustion or dysfunction rather than an absence of T‐cell infiltration.

These collective findings underscore the limitations of ICIs as a standalone treatment for MASLD‐related HCC and point to specific underlying resistance mechanisms. While metabolic dysfunction provides the foundation for this resistance, growing evidence suggests that gut‐liver axis dysregulation acts as a critical accelerant, where distinct dysbiotic signatures and altered metabolite profiles perpetuate chronic inflammation and immune tolerance.

## 5 Mechanism of Resistance to ICIs in MASLD‐Related HCC

The primary driver of ICI resistance in MASLD‐HCC is the aberrant metabolic landscape intrinsic to the disease. The hepatic microenvironment, saturated with accumulated lipids, creates a hostile niche for anti‐tumor immunity. Specifically, the accumulation of long‐chain and very‐long‐chain fatty acids (LCFAs/VLCFAs) triggers lipotoxicity and lipid peroxidation‐driven ferroptosis in CD8+ T cells, directly compromising their cytotoxic capacity and rendering them dysfunctional independent of checkpoint status [48]. Similarly, excessive cholesterol accumulation within the tumor microenvironment activates the endoplasmic reticulum (ER) stress sensor XBP1, which drives the upregulation of exhaustion markers—such as PD‐1 and 2B4—on T‐cell surfaces [49]. Furthermore, dysregulated expression of carnitine palmitoyltransferase (CPT) enhances mitochondrial fatty acid oxidation and reactive oxygen species (ROS) production, leading to the apoptosis and depletion of critical CD4+ T cell subsets [50].

However, these metabolic hurdles do not act in isolation. Patients with MASLD‐HCC face an additional, compounding barrier to efficacy: severe gut dysbiosis that is distinct from and more pronounced than in other HCC etiologies. While metabolic stress initiates T‐cell dysfunction, dysbiosis amplifies this exhaustion through aberrant bacterial metabolite production and systemic immune suppression. This creates a “two‐hit” mechanism of resistance: the metabolic hit establishes a baseline of exhausted immunity, while the dysbiotic hit entrenches and magnifies this state, preventing immune reactivation even with checkpoint blockade. Consequently, the uniquely severe resistance phenotype observed in MASLD‐HCC is the product of this synergy between metabolic toxicity and microbial dysregulation. This review will focus on elucidating the specific role of the gut microbiota as this critical amplifying factor, examining how dysbiosis compounds baseline metabolic exhaustion to sustain the profoundly immunosuppressive environment of MASLD‐HCC.

## 6 Effect of Dysbiosis on ICI Resistance in MASLD‐Related HCC

**FIGURE 4:**
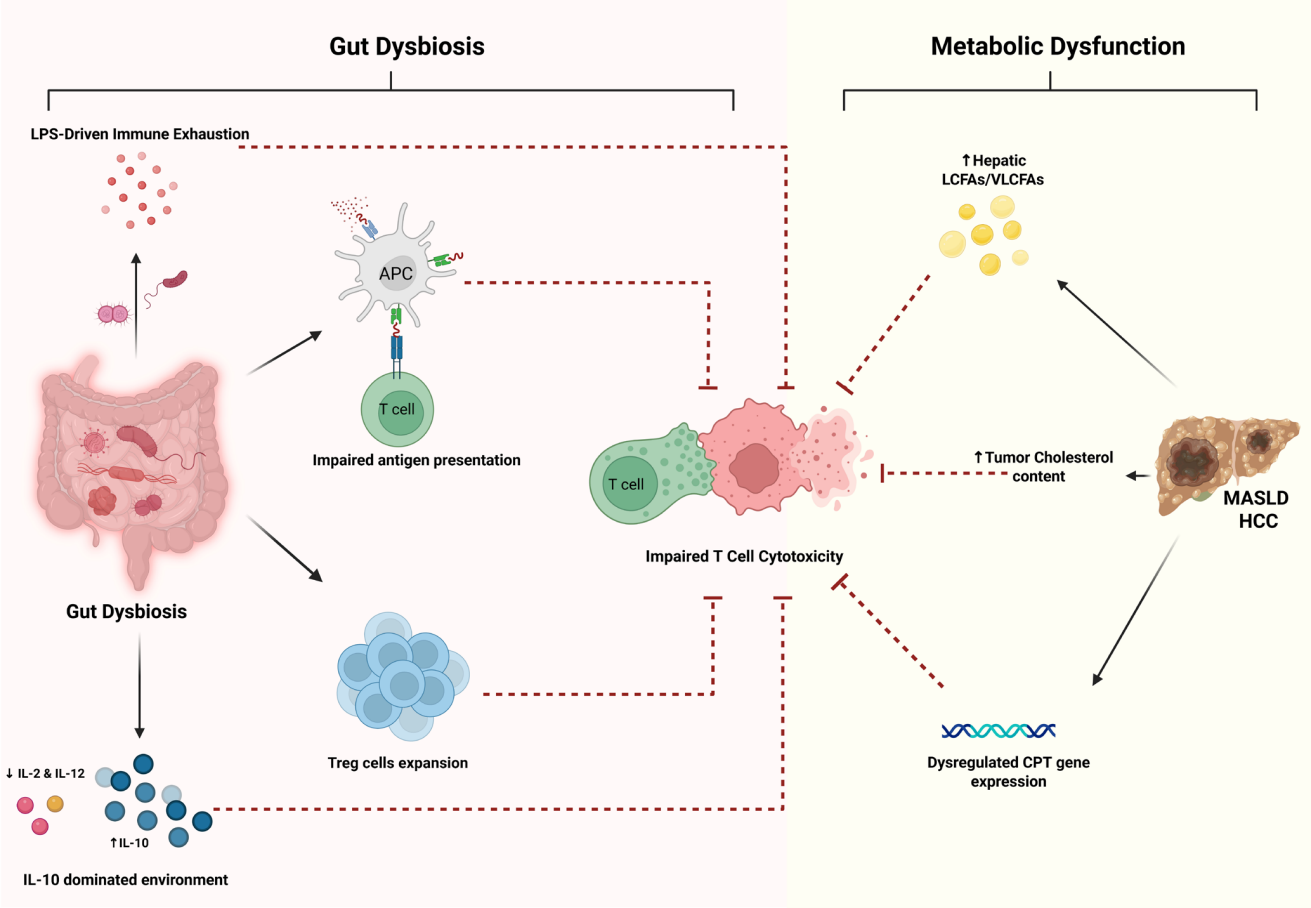
Converging Mechanisms Driving T Cell Exhaustion in MASLD‐HCC: Gut Dysbiosis (pink) and Metabolic Dysfunction (beige). APC, antigen‐presenting cell; CPT, carnitine palmitoyltransferase; HCC, hepatocellular carcinoma; IL, interleukin; LCFAs, long‐chain fatty acids; LPS, lipopolysaccharide; MASLD, metabolic dysfunction–associated steatotic liver disease; Treg, regulatory T cell; VLCFAs, very‐long‐chain fatty acids.

In MASLD‐HCC, gut dysbiosis undermines anti‐tumor immunity through four interconnected mechanisms that operate in parallel to drive T‐cell exhaustion and therapeutic resistance (Figure 4). These pathways—(1) impaired antigen presentation, (2) expansion of immunosuppressive regulatory T cells (Tregs), (3) the creation of an IL‐10‐dominated cytokine environment, and (4) chronic LPS‐driven immune hyperstimulation converge to create a distinct immunometabolic niche where cytotoxic CD8+ T cells are rendered functionally inert. The following sections detail how these mechanisms sequentially integrate to establish robust ICI resistance.

### 6.1 Mechanism 1: Impaired Antigen Presentation

The first critical failure in the anti‐tumor response is a fundamental defect in antigen recognition. Dysbiosis in MASLD‐HCC is strongly associated with the suppression of antigen‐presenting cells (APCs), the gatekeepers of adaptive immunity [16]. This represents the critical first step in the pathway to ICI failure: without effective tumor antigen recognition, even mobilized CD8+ T cells cannot identify and target their cancerous prey. Research indicates that the specific gut microbiota profile of MASLD‐HCC patients correlates with significantly reduced proliferation of key APC populations, including monocytes (*p* = 0.002) and B lymphocytes (*p* = 0.006), compared to patients with MASLD cirrhosis or healthy controls [16]. This deficit impairs the initial priming of CD8+ T cells, meaning that fewer effector cells are activated to identify and target tumor antigens. Consequently, the immune system is effectively “blinded” at the very first step of the cancer‐immunity cycle.

### 6.2 Mechanism 2: Dysbiosis‐Driven Treg Expansion

Compounding this defect in activation is a profound shift toward active suppression. Dysbiosis in MASLD‐HCC produces an aberrant profile of short‐chain fatty acids (SCFAs) that reprograms immune cells away from anti‐tumor activity. Notably, elevated levels of butyrate in the stool of MASLD‐HCC patients correlate positively with the expansion of regulatory T cells (Tregs) (*R* = 0.67, *p* = 0.013) and negatively with cytotoxic CD8+ T cells (*R* = −0.80, *p* = 0.024) [16]. As a result, MASLD‐HCC patients exhibit significantly higher frequencies of Tregs compared to controls (*p* < 0.0001) [16]. These Tregs directly inhibit CD8+ T‐cell function, creating a vicious cycle: the dysbiotic gut limits APC‐mediated T‐cell priming while simultaneously fueling a population of suppressor cells that actively block any residual anti‐tumor activity [51].

### 6.3 Mechanism 3: IL‐10‐Mediated Immunosuppression

The link between APC suppression and Treg expansion is solidified by a dysbiosis‐induced cytokine shift. Bacterial extracts from MASLD‐HCC patients stimulate a significantly higher production of the potent immunosuppressive cytokine IL‐10 (*p* = 0.003) compared to controls [16]. This production is mechanistically driven by microbial metabolites like butyrate, which bind to the GPR109a receptor on dendritic cells and macrophages. This interaction effectively converts APCs from immune activators into “IL‐10 factories” [52].

Elevated IL‐10 serves a dual inhibitory role: it promotes Treg development and drives the exhaustion of intratumoral CD8+ T cells by enhancing N‐glycan branching, which reduces antigen sensitivity [53]. Concurrently, the dysbiotic microbiota fails to stimulate the production of essential pro‐inflammatory cytokines, with significantly lower levels of IL‐2 (*p* = 0.023) and IL‐12 (*p* = 0.001) observed in MASLD‐HCC patients [16]. Since IL‐2 and IL‐12 are vital for T‐cell proliferation and effector function [54, 55, 56], their absence, combined with high IL‐10, creates a “starvation and suppression” environment that ensures T‐cell dysfunction.

### 6.4 Mechanism 4: LPS‐Driven Chronic Immune Hyperstimulation and Exhaustion

Beyond metabolite signaling, dysbiosis promotes exhaustion through chronic, low‐grade inflammation. In MASLD patients, compromised intestinal barrier integrity facilitates the translocation of lipopolysaccharides (LPS) into the portal circulation, a process exacerbated by the depletion of beneficial mucin‐degrading bacteria like *Akkermansia* and *Bifidobacterium* [57, 58]. This continuous exposure to microbial antigens leads to chronic hyperstimulation of immune cells. Paradoxically, this persistent activation does not strengthen immunity but rather accelerates T‐cell exhaustion—a state known as immune tolerance [59]. The result is a population of T cells that are “burnt out” and incapable of mounting a cytotoxic response, further promoting tumor progression [59].

### 6.5 The Integrated Result: CD8 T Cell Exhaustion and ICI Failure

The four dysbiosis‐driven mechanisms above converge on a single critical outcome: exhaustion of CD8+ cytotoxic T cells, a phenomenon characterized by a progressive loss of function and diminished capacity for T cell self‐renewal, acting as a barrier to cellular immunotherapies [60]. This state explains the critical paradox observed in clinical and preclinical studies. For instance, Pfister et al. demonstrated that while MASLD‐HCC tumors are infiltrated by high numbers of CD8 + PD1+ T cells, typically a marker of potential ICI responsiveness, these tumors fail to regress upon anti‐PD1 treatment [43]. In contrast, non‐MASLD tumors with similar infiltrates respond effectively [43]. This disparity definitively proves that the core issue in MASLD‐HCC is not a lack of T cells or checkpoint targets, but rather a deep functional exhaustion driven by the gut‐liver axis. The T cells are present but rendered dysfunctional by the metabolic and microbial environment, meaning that releasing the “brakes” with ICIs is futile because the “engine” (the T cell) is already broken.

## 7 Exploring the Role of Microbiota in Optimizing Response to Immunotherapy for MASLD‐Related HCC Patients

In contrast to the stability of the human genome, the adaptability of the gut microbiota presents a promising opportunity for the development of personalized therapeutic interventions [61]. Recent breakthroughs in molecular techniques, such as high‐throughput sequencing and metagenomics, have enabled researchers to delve deeper into the intricate dynamics of the gut microbiota and their effect on the host [62]. Consequently, the utilization of the microbiome as a potent tool for treating various medical conditions has emerged as a burgeoning field of study. Particularly noteworthy is the potential of microbiome modulation to enhance the efficacy of immunotherapy in cancer treatment.

The following table summarizes key beneficial bacteria and the mechanisms by which they augment immune checkpoint activity (Table 2).

**TABLE 2:** Example of beneficial bacteria and their pathophysiology to enhance ICI response.

Bacteria	Effect on immune cells	Effector metabolite
*Faecalibacterium prausnitzii* [63, 64]	Promotes the growth and specialization of various T cell types, particularly cytotoxic T cells	—
*Lactobacillus reuteri* [65]	Facilitates ICI response	Tryptophan
*B. pseudolongum* and *L. johnsonii* [66] *Bacteroides fragilis and Bacteroides thetaiotaomicron Burkholderiales* [67]	Stimulates IL‐12 and antigen dependent activation of Th1 cells which enhance effectiveness of anti‐CTLA‐4 treatment	Inosine
*Akkermansia muciniphila* [32, 64, 68, 69]	Activates the recruitment of antigen‐presenting cells, natural killer cells, and T helper cells through the secretion of IL‐12 Reduces immunosuppressive myeloid cells (MDSCs, M2 macrophages) and restores intestinal barrier integrity	Propionic acid
*Bifidobacterium bifidum* [70, 71]	Enhances CD8+ T cells proliferation and stimulates the production of interferon‐γ, facilitating the anti‐PD‐1/PD‐L1 efficacy	—

Abbreviations: ICI, immune checkpoint inhibitors; SCFA, short chain fatty acids.

### 7.1 Role of Specific Bacteria in MASLD‐Associated HCC

Emerging evidence highlights that gut microbiota composition profoundly influences both the pathogenesis of MASLD‐HCC and the response to immunotherapy. Specific microbial taxa exert protective or pathogenic effects by modulating lipid metabolism, bile acid signaling, and reprogramming the tumor immune microenvironment.

#### 7.1.1 *Akkermansia muciniphila* as an Immunometabolic Modulator in MASLD‐HCC

Oral administration of *Akkermansia muciniphila* combined with anti‐PD‐1 therapy has been shown to induce maximal tumor suppression, with the synergistic benefit being more pronounced in MASLD‐associated HCC models than in HCC without metabolic dysfunction [32]. This MASLD‐specific efficacy appears to stem from the ability of *A. muciniphila* to attenuate hepatic steatosis and slow disease progression by suppressing cholesterol biosynthesis and reducing multiple bile acid–derived metabolites in the serum [32]. Concurrently, *A. muciniphila* restores intestinal barrier integrity, thereby lowering circulating lipopolysaccharide (LPS) and bile acid levels [32].

These metabolic improvements coincide with marked remodeling of the immune microenvironment. Specifically, *A. muciniphila* reduces intratumoral monocytic myeloid‐derived suppressor cells (MDSCs) and M2 macrophages via inhibition of TLR2–NF‐κB signaling [32]. This shift facilitates the enrichment of effector‐memory CD4^+^ and CD8^+^ T cells and increases the infiltration of PD‐1^+^ CD4^+^/CD8^+^ T cells within tumors [32].

#### 7.1.2 *Bifidobacterium pseudolongum* in MASLD‐HCC

*Bifidobacterium pseudolongum* has been identified as one of the most depleted bacterial species in MASLD‐associated HCC. Oral administration of this bacterium significantly reduced tumor burden in two diet induced MASLD‐HCC mouse models (*p* < 0.01) [72]. Mechanistically, *B. pseudolongum* produces acetate, which reaches the liver via the portal circulation and activates GPR43 on hepatocytes, thereby suppressing the oncogenic IL‐6/JAK1/STAT3 signaling cascade that drives MASLD‐HCC progression [72].

These mechanistic findings were validated in human MASLDHCC cell lines, where conditioned medium from both‐ *B. pseudolongum* and acetate inhibited cell proliferation, induced G1/S phase cell cycle arrest and apoptosis, and suppressed IL‐6/JAK1/STAT3 signaling [72].

### 7.2 Role of FMT

Fecal microbiota transplantation (FMT) involves transferring fecal material from a healthy donor to a recipient with disrupted gut microbiota, with the primary goal of restoring microbial balance [73]. The procedure can be administered through various routes, including colonoscopy, capsules, or enemas [74]. By introducing a diverse community of microorganisms to the recipient's gut, FMT helps reestablish a healthy microbial ecosystem [75]. While the precise mechanisms underlying FMT effectiveness remain under investigation, current evidence suggests it operates through multiple pathways, including competitive exclusion of pathogenic bacteria, immune system modulation, and production of antimicrobial substances by the transplanted microorganisms.

#### 7.2.1 FMT and Immunotherapy in Cancer Treatment

Building on its therapeutic potential, FMT has emerged as a promising strategy for enhancing immunotherapy responses in cancer treatment [76]. Multiple research studies have demonstrated that FMT can serve as an adjunctive therapy to augment the efficacy of ICI therapy [77, 78].

Preclinical evidence supports this application, with studies showing that FMT can activate immune responses to anti‐PD‐L1 therapy in mouse melanoma models [79]. Similarly, FMT has been shown to improve anti‐tumor immune responses while reducing immunosuppressive T regulatory cells in mice with colorectal cancer and rats with primary liver cancer [80].

Translating these findings to clinical settings, researchers have successfully overcome initial resistance to anti‐PD‐1 immune checkpoint inhibitors in patients with refractory melanoma by administering FMT from treatment responders [77]. Following FMT, these previously resistant patients exhibited increased proportions of activated CD8+ T cells with enhanced cytotoxic capabilities [77]. Additionally, responders demonstrated reduced levels of several immune‐suppressing cytokines, particularly IL‐8, which has been associated with poor prognosis in cancer patients receiving ICIs across various malignancies [77, 81].

#### 7.2.2 FMT in Mitigating Immunotherapy‐Related Adverse Effects

Beyond enhancing treatment efficacy, the microbiome also plays a crucial role in potentially reducing the adverse effects associated with immunotherapy [82]. FMT has demonstrated remarkable effectiveness in managing ICI‐induced colitis, one of the most common immune‐related adverse events [83]. The following table summarizes clinical trials investigating FMT's dual role in both enhancing ICI effectiveness and combating treatment‐related side effects in cancer patients (Table 3).

**TABLE 3:** Clinical trials investigating the effects of FMT on ICI therapy in different cancers.

Study identifier	Enrolled patients	Cancer type	ICI type	Any combined medication	FMT source	FMT procedure	Results	Safety profile	Antibiotic use before FMT
*Studies designed to measure safety and therapeutic effectiveness of FMT on ICIs*									
NCT04116775	32	Prostate	Pembrolizumab	Enzalutamide	Pembrolizumab/Enzalutamide treatment responders	Endoscopy	—	—	No
NCT03353402	40	Melanoma	Anti‐PD‐1	No	ICI treatment responders	Colonoscopy then capsules	ORR 30%; all responders from single donor; increased intratumoral CD8+ T cells	No grade 2–4 irAEs; 1 mild GI toxicity (bloating)	—
NCT03772899	20	Melanoma	Pembrolizumab or Nivolumab	No	Healthy donors	Capsules	ORR 65% (13/20; 4 CR, 9 PR); mPFS 52.8 months (with FMT‐AE) versus 15.9 months (without); mOS 52.8 mo; 1‐year OS 95%, 3‐year OS 53%	Grade 3: 5 pts. (25%); No grade 4–5 irAEs; Safe	—
NCT05251389	24	Melanoma	Anti‐PD‐1	No	ICI treatment responders and non‐responders	Endoscopy	—	—	Vancomycin
NCT06030037	56	Melanoma	Pembrolizumab	Lenvatinib	ICIs treatment Responders	Colonoscopy then capsules	—	—	No
NCT03341143	18	Melanoma	Pembrolizumab	No	ICI treatment responders	Colonoscopy	ORR ≥ 33%; Clinical benefit 40% (6/15); 1 CR, 2 PR, 3 SD; mPFS 3.0 months overall; mPFS 14.0 months in responders	Safe; minimal grade 1–2 AEs; No grade 3+ irAEs	No
NCT04951583	45	Melanoma/NSCLC	Any ICIs	No	—	Capsules	NSCLC: ORR 80% (16/20 PR); DCR 95%; 1‐year PFS 65%, 1‐year OS 100% Melanoma: ORR 75% (4 CR, 11 PR); 1‐year PFS 58%, 1‐year OS 79%	NSCLC: No grade 3+ AEs Melanoma: 65% grade 3+ irAEs	No
NCT05502913	80	Lung	—	Chemotherapy	ICI treatment responders	Capsule	—	—	Rifaximin
NCT05008861	20	Lung	Anti‐PD‐1/PD‐L1	Platinum based chemotherapy	—	Capsule	ORR 44%; 93.3% of FMTs administered without dose‐limiting toxicities	irAEs in 80% patients; 93.3% tolerability rate	No
NCT05669846	26	Lung	Pembrolizumab	No	ICI treatment responders	Colonoscopy/sigmoidoscopy	ORR 80%, DCR 95% in treatment‐naïve NSCLC cohort, 1‐year PFS 65%; 1‐year OS 100%	Safe; only grade 1 FMT‐related AEs	No
NCT04924374	20	Lung	Pembrolizumab, Nivolumab, Atezolizumab	No	—	Capsule	—	Well tolerated; minimal AE	No
NCT04130763	10	GIT	Anti‐PD‐1 Therapy	No	Healthy individuals	Capsule	ORR 12.5% (1/8 PR); Clinical benefit 50% (1 PR + 3 SD > 6 months)	Well tolerated; No serious AEs	No
NCT05279677	30	GIT	Sintilimab	Fruquintinib	—	Capsule	—	—	No
NCT05001360	—	GIT	Nivolumab	No	—	Capsule	ORR 7.7% (1/13 PR); DCR 46.2% (1 PR + 5 SD, 1 HCC patient showed maximum tumor reduction of 47.7%)	Well tolerated, FMT‐related adverse events: Minimal	No
NCT04729322	15	GIT	Pembrolizumab or Nivolumab	No	ICI treatment responders	Colonoscopy then capsules	—	—	Metronidazole + Vancomycin + Neomycin
NCT05690048	48	HCC	Atezolizumab	Bevacizumab	—	Capsule	—	—	Vancomycin oral
NCT05750030	12	HCC	Atezolizumab	Bevacizumab	ICI treatment responders	—	—	—	No
NCT04758507	50	RCC	Any ICIs	No	—	Colonoscopy then capsules	12‐months PFS 70% (d‐FMT) versus 41% (p‐FMT, *p* = 0.053); mPFS 24.0 versus 9.0 months (*p* = 0.028); ORR 52% versus 32%; Median OS 41 versus 28.3 months	Grade 3+ AEs 28% (d‐FMT) vs. 16% (p‐FMT)	No
NCT04521075	42	Multiple types	Nivolumab	No	ICI treatment responders	Capsule ± colonoscopy	ORR 30% (interim); 1 CR reported	Well tolerated; manageable AEs	No
NCT05533983	50	Multiple types	Nivolumab	No	—	—	—	—	No
*Studies designed to investigate FMT mitigating immunotherapy related adverse effects*									
NCT06206707	20	Melanoma and/or Renal	Any ICIs	No	—	Capsule	—	—	No
NCT04038619	40	Multiple types	—	Loperamide	—	Colonoscopy	Interim (*n* = 7): 71.4% symptom improvement within 1 day; 83.3% colitis remission; 57.1% resumed immunotherapy	Safe; only transient fever/cramping (42.8%)	No
NCT03819296	800	Multiple types	Any ICIs	No	—	—	92% clinical remission of grade 3+ IMC in 12‐patient case series	Safe; well tolerated in 12‐pt case series	—

Abbreviations: AE, adverse event; CR, complete response; d‐FMT, donor‐derived FMT; DCR, disease control rate; FMT, fecal microbiota transplantation; HCC, hepatocellular carcinoma; ICI, immune checkpoint inhibitors; irAE, immune‐related adverse event; mOS, median overall survival; mPFS, median progression‐free survival; NSCLC, non‐small‐cell lung cancer; ORR, objective response rate; OS, overall survival; p‐FMT, placebo FMT; PR, partial response; RCC, renal cell carcinoma; SD, stable disease.

#### 7.2.3 FMT in MASLD‐Related HCC Patients

FMT has been safely applied to patients with MASLD, with studies demonstrating its ability to reverse dysbiosis‐mediated immunosuppression through multiple mechanisms. In a randomized controlled clinical trial, MASLD patients receiving FMT showed improvement in abnormal small intestinal permeability, indicating restoration of the gut barrier [84]. This finding is particularly significant because disrupted gut permeability facilitates the translocation of lipopolysaccharides and other microbial toxins and metabolites into the portal and systemic circulation, which subsequently promotes chronic inflammation, immune exhaustion, and an immunosuppressive tumor microenvironment in MASLD‐HCC patients [57].

By reducing this “leaky gut” phenotype, FMT has the potential to decrease endotoxin load and normalize immune tone in the liver. Consistent with this mechanism, patients with MASLD who received FMT demonstrated lower levels of circulating bacterial metabolites and an improved inflammatory profile, suggesting a shift from a pro‐tumorigenic, immunosuppressive milieu toward a more immune‐permissive state [85]. Beyond these immunomodulatory effects, FMT has also shown promise in ameliorating the underlying fatty liver disease by restoring gut microbiota balance [86]. This restoration may indirectly reduce HCC risk and enhance responsiveness to systemic therapies in MASLD‐HCC by attenuating lipotoxicity, insulin resistance, and metabolic inflammation.

Collectively, these observations support FMT as a rational, though still investigational, adjunctive approach in MASLD‐related HCC aimed at correcting upstream microbiota‐driven drivers of immune dysfunction. However, future studies are needed to determine whether these mechanistic improvements in gut permeability, metabolic markers, and inflammatory profiles translate into enhanced cancer treatment outcomes, such as tumor response, progression‐free survival, or overall survival in MASLD‐related HCC patients.

While the therapeutic potential of FMT and other microbiota‐modulating interventions is promising, several logistical challenges must be addressed before widespread clinical implementation (Table 4). A primary concern is the risk of transmitting multi‐drug resistant bacteria, viruses, or fungi through FMT [87, 94]. This risk necessitates rigorous mitigation strategies, including thorough screening of donor feces and virus polymerase chain reaction testing on fecal samples prior to transplantation [95]. Furthermore, establishing reliable stool banks for FMT has proven challenging, which may hinder the delivery of FMT therapy to MASLD‐HCC patients who could benefit from this intervention [95].

Table 4 summarizes the key risks associated with FMT, as identified in the current literature.

**TABLE 4:** Fecal microbial transplantation risk categories and safety considerations.

Risk category	Description	Examples/Notes
Antibiotic‐resistant bacteria	Transmission of drug‐resistant bacterial strains that may lead to severe, difficult‐to‐treat systemic infections	Drug‐resistant *E. coli* bacteremia ESBL‐producing *E. coli* causing fatal infections [87, 88, 89]
Infectious pathogens	Transfer of pathogenic organisms, posing elevated risks particularly in immunocompromised recipients	Enteropathogenic *E. coli* (EPEC) and Shiga toxin‐producing *E. coli* (STEC) infections Rare instances of intestinal perforation and mortality [89, 90, 91]
Long‐term ecological impacts	Theoretical risks of inducing non‐infectious conditions through durable alterations of the recipient's gut microbiota	Potential induction of obesity, metabolic disorders, or carcinogenesis *Note:* While theoretically possible, no definitive long‐term harm signal has been established to date [89]
Donor screening limitations	The necessity for rigorous screening protocols creates supply constraints due to high donor disqualification rates	Donor rejection rates can reach up to 97% Centralized, standardized stool banks are essential to maintain safety and supply stability [92]
Regulatory alerts	Official warnings regarding safety protocols and serious adverse events	FDA alerts (2019, 2020) emphasizing the critical need for screening against multi‐drug resistant organisms and specific pathogens [93]

Abbreviation: ESBL, extended spectrum beta‐lactamase.

### 7.3 Role of Probiotics

Probiotics consist of living microbes that, when administered in adequate amounts, confer a health benefit on the host. These beneficial microbes play a critical role in regulating physiological functions and disease processes, primarily by modulating systemic inflammation, enhancing immune surveillance, and maintaining the homeostatic balance of the intestinal microbiota [96].

Emerging evidence suggests that probiotics interact intricately with the host immune system, potentially influencing the therapeutic efficacy of cancer treatments. The pivotal relationship between the gut environment and immune function was highlighted by Spencer and colleagues, who demonstrated that low‐fiber diets compromise interferon‐gamma (IFN‐γ) production and cytotoxic T‐cell responses [97]. These findings underscore the rationale for microbiome‐targeted interventions, such as probiotic supplementation, to optimize the host response to ICI therapy [97]. Consistent with this hypothesis, preclinical research utilizing murine tumor models has shown that the administration of *Bifidobacterium pseudolongum* , *Lactobacillus johnsonii* , and *Olsenella* species stimulates potent anti‐tumor immune responses and enhances the effectiveness of ICIs [66, 70, 72].

Translating these preclinical insights into clinical practice, several studies have corroborated the therapeutic potential of probiotics in human cohorts. For instance, a 2021 study in Japan reported positive clinical outcomes in patients with advanced or recurrent non‐small cell lung cancer (NSCLC) treated with anti‐PD‐1 monotherapy, revealing a significant correlation between probiotic administration and extended progression‐free survival [98]. Similarly, recent data has established a link between probiotic usage and improved efficacy of nivolumab across various malignancies, including gastric cancer [99]. Providing higher‐level evidence, a meta‐analysis of five studies encompassing 1031 NSCLC patients further validated these observations [100]. The analysis revealed that probiotic administration was associated with significantly improved overall survival (HR = 0.50, 95% CI: 0.30–0.85, *p* = 0.01) and progression‐free survival (HR = 0.51, 95% CI: 0.42–0.61, *p* < 0.01) [100].

#### 7.3.1 Probiotics in MASLD‐Related HCC Patients

Although clinical trials specifically testing probiotics in MASLD‐HCC are limited, the therapeutic application of *Akkermansia muciniphila* represents a leading candidate for intervention. Building on the immunometabolic mechanisms previously discussed, recent translational research highlights the significant potential of this bacterium. In orthotopic mouse models, daily oral supplementation of *A. muciniphila* went beyond merely altering immune markers to produce tangible therapeutic synergy with anti‐PD‐1 agents, resulting in maximal tumor growth suppression [32].

These preclinical successes are strongly supported by parallel clinical observations. Research indicates that patients with MASLD‐HCC exhibit a significantly reduced abundance of *A. muciniphila* compared to healthy controls, suggesting a critical microbial deficit [32]. Crucially, restoring or maintaining higher baseline levels of this bacterium correlates with improved responsiveness to PD‐1 blockade and extended progression‐free survival [32]. This relationship is quantified by striking response data: patients with high baseline abundance achieved objective response rates of 41%, compared to only 7% in those with low abundance [32]. Consequently, probiotic strategies aimed at repopulating *A. muciniphila* represent a rational and promising approach to overcome primary resistance to checkpoint inhibitors in this distinct patient subset.

### 7.4 Role of Antibiotics

The modulation of ICI efficacy by antibiotics remains a subject of intense debate within the oncology community [101]. Preclinical evidence suggests a detrimental effect; murine tumor models treated with broad‐spectrum antibiotics consistently demonstrate resistance to ICI therapy [67, 68]. These findings are corroborated by numerous clinical studies indicating that antibiotic‐induced dysbiosis negatively impacts patient outcomes. Specifically, the concurrent use of antibiotics and ICIs has been associated with poor prognosis, including reduced progression‐free and overall survival, across various malignancies such as advanced renal cell carcinoma, non‐small cell lung cancer, melanoma, and urothelial carcinoma [102, 103, 104, 105].

Innovative strategies to mitigate this risk are emerging. For instance, a recent randomized clinical trial demonstrated that DAV132, a novel colon‐targeting adsorbent, effectively prevented antibiotic‐induced dysbiosis when co‐administered with antibiotics, thereby preserving the efficacy of immunotherapy [106]. However, the relationship between antibiotics and ICI response is not uniformly negative. A substantial international cohort of 450 patients with HCC revealed a paradoxical finding: antibiotic use within 30 days of ICI therapy was associated with *improved* immunotherapy outcomes, independent of other clinical characteristics [107]. This divergence underscores the complexity of the interplay between microbial modulation, host immunity, and therapeutic efficacy, suggesting that the impact of antibiotics may be context‐dependent or tumor‐specific.

## 8 Glimpse Into the Future

### 8.1 Synthetic Microbial Consortia

Synthetic microbial consortia represent a precision medicine approach to microbiome modulation. This technique involves the isolation and culture of specific bacterial strains from healthy donors, which are then combined into a defined mixture designed to perform distinct therapeutic functions in the recipient [108]. Unlike traditional FMT, this method eliminates the risk of transferring uncharacterized or potentially pathogenic organisms.

The therapeutic potential of this approach is highlighted by recent preclinical findings. Kwan et al. [109] identified a specific signature of eight bacterial species depleted in patients with MASLD‐associated fibrosis. When this defined consortium was administered to germ‐free mice fed a methionine‐ and choline‐deficient diet (a standard model of MASLD‐induced fibrosis), it significantly ameliorated liver fibrosis, steatosis, and injury markers [109]. Beyond metabolic improvements, synthetic consortia have also been shown to promote the expansion of interferon‐γ–producing CD8+ T cells within the intestinal mucosa, thereby potentiating anti‐tumor immunity [110].

While these results are encouraging, moving from the lab to the clinic is just beginning. A Phase I trial (MET4‐IO) evaluating a synthetic consortium in combination with ICIs for advanced solid tumors confirmed safety and tolerability, though efficacy signals remain preliminary [111]. Most importantly, we have yet to see any human trials testing these consortia specifically in patients with MASLD or MASLD‐related liver cancer. Before this approach can become a viable treatment, we need to answer some difficult questions, including the ability of inoculated strains to stably engraft within a dysbiotic liver disease environment, their interaction with the native commensal flora, and their safety profile in patients with decompensated cirrhosis and portal hypertension. Consequently, synthetic consortia remain an investigational strategy requiring rigorous validation in well‐controlled human trials.

### 8.2 Phage Therapy

Bacteriophage therapy, the use of viruses to specifically target and eliminate pathogenic bacteria, offers a highly selective method for correcting dysbiosis [112]. In the context of liver disease, preclinical studies have demonstrated the efficacy of phages in eradicating specific pathobionts, such as cytolysin‐producing *Enterococcus faecalis* in alcoholic liver disease [113], and alcohol‐producing *Klebsiella pneumoniae* in MASLD models [114]. These interventions have successfully reduced bacterial burden and hepatic inflammation in small animal studies.

Despite this potential, significant hurdles impede clinical application. Challenges include the unintended disruption of non‐target bacterial species [115], the rapid emergence of phage‐resistant bacterial strains [116], and the potential for phages themselves to induce intestinal inflammation or colitis [117]. To date, no clinical trials have evaluated phage therapy in metabolic liver diseases. Therefore, while phage therapy represents a promising tool for precision microbiome editing, extensive safety and efficacy testing is required to determine its viability for patients with MASLD and MASLD‐HCC.

## 9 Conclusion

The gut microbiota has emerged as a critical orchestrator in the progression of MASLD to liver cirrhosis and HCC, serving as both a key pathogenic driver and a promising therapeutic target, particularly for patients receiving immune checkpoint inhibitor therapy. Moving forward, research efforts should prioritize the investigation of specific bacterial candidates with demonstrated therapeutic potential, including *Mediterraneibacter gnavus* [23], *Akkermansia muciniphila* [32, 68], *Faecalibacterium prausnitzii* [68], and *Bifidobacterium species* [67]. Future investigations must evolve beyond observational associations to rigorous, large‐scale, multi‐center clinical trials that stratify patients by underlying liver disease etiology. Such comprehensive data will be instrumental in decoding the complex, context‐specific host–microbe interactions in liver disease, ultimately paving the way for the development of individualized treatment plans tailored to each patient's unique gut microbial profile.

## Author Contributions

**Mazen Elsheikh:** investigation (lead), methodology (lead), writing – original draft (lead), writing – review and editing (lead). **Mohamad Ali Ibrahim:** investigation (supporting), methodology (supporting), writing – review and editing (supporting). **Sherry Fares:** methodology (supporting), writing – review and editing (supporting). **Megha Bhongade:** methodology (supporting), writing – original draft (supporting). **Karim Adhem:** methodology (supporting), writing – review and editing (supporting). **Ximena I. Ramirez‐Morales:** methodology (supporting), writing – review and editing (supporting). **Ahmed O. Kaseb:** investigation (supporting), supervision (supporting). **Joseph Petrosino:** investigation (supporting), supervision (supporting). **Manal M. Hassan:** methodology (lead), supervision (lead). **Prasun K. Jalal:** investigation (lead), methodology (lead), supervision (lead).

## Conflicts of Interest

The authors declare no conflicts of interest.

## Data Availability

The authors have nothing to report.
